# Felis Punctatis: Cat Claw-induced Punctures

**DOI:** 10.7759/cureus.1927

**Published:** 2017-12-08

**Authors:** Philip R Cohen, Douglas S Ramsay

**Affiliations:** 1 Department of Dermatology, University of California, San Diego; 2 Department of Oral Health Sciences, University of Washington, Seattle, WA

**Keywords:** cat, claw, feline, felis, kneading, punctatis, punctures

## Abstract

Animal-induced conditions in humans predominantly present as infectious zoonoses. However, trauma-associated injuries from the teeth or claws can also occur. Several zoonotic infections can be transmitted by cats, a common household pet, to their owners. The clinical features of a woman who developed multiple sites of trauma-induced cutaneous punctures from her cat’s paws while it was kneading on her clothes-covered abdomen are described. The repetitive insertion and withdrawal of the sharp tips of the cat’s claws created distinctive groups of erythematous punctures on the patient’s skin. We suggest that Latin nomenclature be used to designate the name for this claw-induced dermatosis that includes not only the causative animal (felis for cat) but also a descriptive term for the skin lesions (punctatis for punctures): felis punctatis.

## Introduction

Domestic cats are common household pets. Several zoonotic conditions have been associated with cats, such as Bartonellosis (cat scratch fever) and sporotrichosis. We describe a woman with abdominal skin punctures created by her cat’s claws during kneading.

## Case presentation

A healthy, afebrile 56-year-old woman presented for evaluation of new asymptomatic red skin lesions on her abdomen. The lesions had appeared in a cluster several days prior and had become redder and more prominent. There were no other cutaneous or mucosal findings.

**Figure 1:**
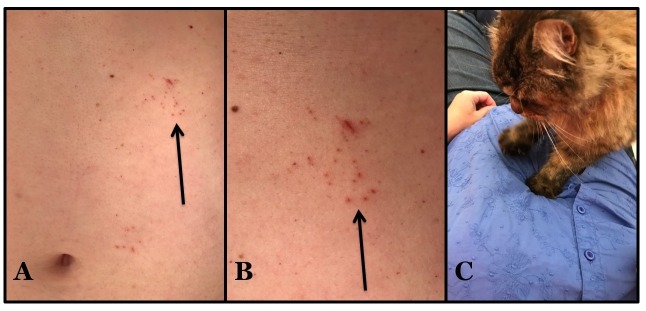
Cat claw-induced punctures: felis punctatis Distant (A) and closer (B) view of the upper left abdomen just below the rib cage of a 56-year-old woman shows groups of individual asymptomatic red punctures and a few excoriations (arrows). The cutaneous lesions were caused by her cat (a domestic long-hair breed) piercing her overlying clothing and skin beneath with the tips of its sharp claws while kneading (C).

Cutaneous examination showed groups of solitary erythematous punctures on the upper abdomen, in an area lateral to and above the umbilicus. An occasional linear excoriation was also noted. Similar lesions were absent on the rest of her body (Figure 1).

Additional history revealed that Bella—a nine pound spayed (long-haired domestic female cat)–stood on her abdomen one evening; specifically, when she was lying on the couch—horizontally on her back with her clothed abdomen exposed—her cat arrived and began to knead. The cat’s claws extended from its paw, pierced the clothing and the underlying skin, and were then withdrawn. This activity was mildly aversive to the patient and was repeated several times by the cat as shown in the linked video (Video 1).

**Video 1:** Felis punctatis (cat claw-induced punctures) The patient’s cat created the punctures of the abdominal skin. The cat repeatedly extended its claws and pressed them through not only the patient’s clothing, but also the skin of her abdomen during the process of kneading.

Topical mupirocin 2% ointment, applied three times daily to the skin punctures, would be an appropriate treatment to prevent a secondary bacterial infection although the patient reported not using the ointment. All of the punctures healed and disappeared within three weeks. However, new sets of lesions occasionally occur since the patient still permits her cat to knead on her abdomen.

## Discussion

Zoonoses are diseases that can be transmitted from animals to people. Cat-related zoonotic conditions can be associated with bacteria, dermatophytes, fleas, fungi, mites, mycobacteria, nematodes, protozoa, ticks, and viruses (Table 1) [1-10]; whether the cat is a mostly indoor or a mostly outdoor animal probably makes a difference in the likelihood of becoming a vector in the transmission of infectious organisms. Our patient presented with a distinctive cat-induced skin condition consisting of numerous punctures and small excoriations in a clustered appearance that, to the best of our knowledge, has not been previously named.

**Table 1:** Causes of cat-associated zoonotic conditions

Causes of cat-associated zoonotic conditions
Bacteria
Bacteroides species
Bartonella henselae (also known as Bartonellosis or cat scratch fever)
Bordetella bronchiseptica
Brucella abortus
Campylobacter jejuni
Capnocytophaga canimorsus
Fusobacterium species
Leptospirosis
Pasteurella multocida
Porphyromonas species
Salmonella species
Staphylococcus species
Streptococcus species
Dermatophyte
Microsporum canis
External parasites
Fleas (bartonellosis, endemic—also known as murine—typhus, and yersiniosis)
Mites (cheyletiella and scabies—also known as mange)
Ticks (ehrlichiosis, Lyme disease, rocky mountain spotted fever and tularemia)
Fungus
Histoplasmosis
Sporotrichosis
Mycobacteria
Mycobacteria species
Nematode
Ancylostoma braziliense (a hookworm causing cutaneous larva migrans)
Toxocara cati (a roundworm causing visceral larva migrans)
Protozoa
Cryptosporitium parvum
Giardia lambia
Toxoplasma gondii
Virus
Rabies

Kneading is the term used to describe the rhythmic motion that cats make with their paws when they repeatedly push them in and out of a soft object; often insertion and withdrawal of extended claws into the pliable object accompanies the repetitive and steady movement of the cat’s paws. Several explanations for the cat’s kneading behavior have been proposed: as an instinctive trait of newborn kittens to stimulate milk flow from the nipples on their mother’s abdomen, to prepare an area for sleep or delivery similar to wild cats patting down tall grass and fallen leaves, to establish a territorial marker of an area since the scent glands in their soft paws release scent on to the site of kneading, and as a signal from female cats—when going into heat—to males that they are willing and able to mate. To prevent cutaneous injury, such as skin punctures in this patient, the cat’s claws can be kept trim by clipping the sharp curved tip of each nail; alternatively, nail guards can be used. The patient also observed that skin punctures are more likely to occur when wearing a thin shirt as opposed to a thick shirt.

Kneading is speculated as the route of feline-transmitted sporotrichosis in a 41-year-old woman in California. Her cats would engage in kneading behavior on her lap and in bed with her. She developed culture-confirmed lymphocutaneous sporotrichosis that presented as a single painless erythematous papule on her left chest wall, followed by the appearance of additional papules in the same area, which subsequently ulcerated over three weeks. Her infection completely resolved, without recurrence during a three-month oral course of itraconazole 200 mg twice daily. Prior to the onset of her illness, her male cat had developed an infection of its right paw; periodic acid-Schiff stain of the biopsy from the infected paw obtained by the veterinarian showed yeast forms consistent with Sporotrichosis schenkii [6].

The cat’s claws’ sharp tips were able to reach the patient’s abdomen through her clothing and create small skin punctures at their site of contact. Mild pain was associated with the acquisition of her lesions. All of the patient’s lesions resolved without any complications; however, since she would still allow her cat to knead on her abdomen—without any intervention to its claws—recurrence of the skin punctures has periodically occurred. We suggest that the dermatosis created by cutaneous punctures in humans caused by cat claws be referred to using the Latin translation of cat puntures: felis punctatis.

## Conclusions

Cat-induced conditions occurring in humans are predominantly infectious zoonoses. In affected individuals, they can be acquired by several routes including direct fur-to-skin contact, fecal-to-oral transmission, inhalation, soil-borne spread, vector-borne spread, and trauma from bites, scratches or punctures. However, kneading-associated localized groups of skin punctures from cat’s claws—a trauma-associated injury—has a distinctive morphologic presentation for which the insightful clinician can confirm the diagnosis by obtaining an appropriate history from the patient. Topical antimicrobial treatment may be suggested to prevent secondary bacterial infection until the lesions heal. Claw clipping to keep the nails trim or nail guards may be instituted to prevent recurrences. We suggest that the name for this claw-induced dermatosis be designated by the Latin translation of words that include not only the causative animal (felis for cat) but also a descriptive term for the skin lesions (punctatis for punctures): felis punctatis.
